# Molecular annotation of ketol-acid reductoisomerases from *Streptomyces* reveals a novel amino acid biosynthesis interlock mediated by enzyme promiscuity

**DOI:** 10.1111/1751-7915.12175

**Published:** 2014-10-09

**Authors:** Karina Verdel-Aranda, Susana T López-Cortina, David A Hodgson, Francisco Barona-Gómez

**Affiliations:** 1 Evolution of Metabolic Diversity Laboratory, Unidad de Genómica Avanzada (Langebio), Cinvestav-IPNKm 9.6 Libramiento Norte, Irapuato, Guanajuato, CP36822, México; 2 Facultad de Ciencias Químicas, Universidad Autónoma de Nuevo LeónSan Nicolás de los Garza, Nuevo León, México; 3 School of Life Sciences, University WarwickCoventry, UK

## Abstract

The 6-phosphogluconate dehydrogenase superfamily oxidize and reduce a wide range of substrates, making their functional annotation challenging. Ketol-acid reductoisomerase (KARI), encoded by the *ilvC* gene in branched-chain amino acids biosynthesis, is a promiscuous reductase enzyme within this superfamily. Here, we obtain steady-state enzyme kinetic parameters for 10 IlvC homologues from the genera *Streptomyces* and *Corynebacterium*, upon eight selected chemically diverse substrates, including some not normally recognized by enzymes of this superfamily. This biochemical data suggested a *Streptomyces* biosynthetic interlock between proline and the branched-chain amino acids, mediated by enzyme substrate promiscuity, which was confirmed via mutagenesis and complementation analyses of the *proC*, *ilvC1* and *ilvC2* genes in *Streptomyces coelicolor*. Moreover, both *ilvC* orthologues and paralogues were analysed, such that the relationship between gene duplication and functional diversification could be explored. The KARI paralogues present in *S. coelicolor* and *Streptomyces lividans*, despite their conserved high sequence identity (97%), were shown to be more promiscuous, suggesting a recent functional diversification. In contrast, the KARI paralogue from *Streptomyces viridifaciens* showed selectivity towards the synthesis of valine precursors, explaining its recruitment within the biosynthetic gene cluster of valanimycin*.* These results allowed us to assess substrate promiscuity indices as a tool to annotate new molecular functions with metabolic implications.

## Introduction

It is well acknowledged that enzymes can be promiscuous or multifunctional, catalysing different chemical transformations upon one or more substrates, or catalysing identical reactions using several related or unrelated substrates (O'Brien and Herschlag, 1999; Khersonsky *et al*., 2006; Khersonsky and Tawfik, 2010). Since the recognition of this phenomenon, enzyme promiscuity has been defined as the ability of enzymes to exert other activities beyond those for which they have evolved, implying that such activities are overall not relevant for the physiology of the organism (Copley, 2003; Khersonsky *et al*., 2006; Khersonsky and Tawfik, 2010). It has also been hypothesized that promiscuous enzymatic activities serve as evolutionary starting points for the appearance of new functions (Jensen, 1976; Khersonsky *et al*., 2006; Piatigorsky, 2007; Khersonsky and Tawfik, 2010). After duplication, for instance, of a metabolic gene encoding for a promiscuous enzyme, subsequent mutations in one of the paralogue could lead to a novel function (Ohno, 1970). Indeed, enzyme promiscuity followed by enzyme recruitment, seems to have given rise to many peripheral metabolic pathways, such as natural products biosynthetic pathways (Vining, 1992).

It could be argued that one of the main challenges in molecular biology is the correct functional annotation of proteins, a situation that is accentuated in the context of promiscuous enzymes. This challenge has been addressed either by reductionist studies focusing in a single protein, or after high-throughput systems-level analyses involving many proteins (Laskowski *et al*., 2005; Redfern *et al*., 2009; Schnoes *et al*., 2013). The trade-off between these approaches represents a conundrum for annotation of enzyme superfamilies, defined as structurally and functionally related enzymes that can catalyse similar reactions upon quite different substrates (Gerlt and Babbitt, 2001; Gerlt *et al*., 2012). Unfortunately, a deep understanding of the relationships among sequence, structure and function of enzyme superfamilies is limited to few cases, such as the enolase superfamily (Gerlt *et al*., 2012).

**Figure 1:**
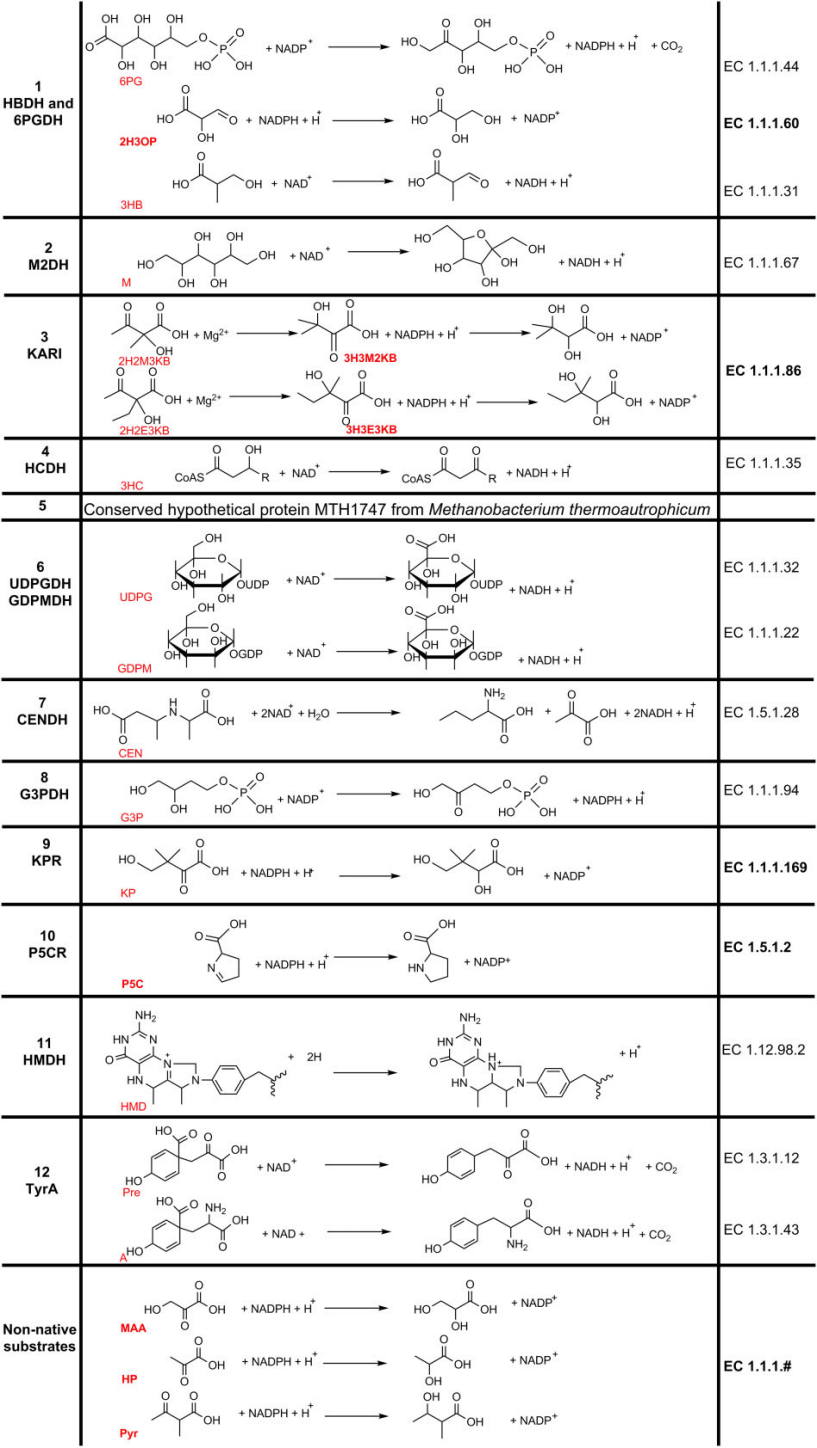
Enzyme members of the 6PGDH superfamily in SCOP. 1: Hydroxyisobutyrate-6-phosphogluconate dehydrogenase (HBDH-6PGDH); 6-phosphogluconate dehydrogenase (6PGDH, EC 1.1.1.44), 2-hydroxy-3-oxopropionate reductase (2H3OPR, EC 1.1.1.60), 3-hydroxyisobutyrate dehydrogenase (3HBDH, EC 1.1.1.31). 2: Mannitol 2-dehydrogenase (M2DH, EC 1.1.1.67). 3: Ketol-acid reductoisomerase (KARI, EC 1.1.1.86). 4: 3-hydroxyacyl-CoA dehydrogenase (3HCDH, EC 1.1.1.35). 5: Conserved hypothetical protein MTH1747. 6: uridine diphosphate (UDP)-glucose/GDP-mannose dehydrogenase (UDPGDH/GDPMDH, EC 1.1.1.22/EC 1.1.1.132). 7: N-(1-D-carboxylethyl)-L-norvaline dehydrogenase (CENDH, EC 1.5.1.28). 8: Glycerol-3-phosphate dehydrogenase (G3PDH, EC 1.1.1.94). 9: Ketopantoate reductase (KPR, EC1.1.1.169). 10: Pyrroline-5-carboxylate reductase (P5CR, EC 1.5.1.2.). 11: 10-methenyltetrahydromethanopterin hydrogenase (HMD, EC 1.12.98.2). 12: TyrA, prephenate dehydrogenase/arogenate dehydrogenase (PreDH/ADH, EC 1.3.1.12/EC 1.3.1.43). The EC numbers of reductases are shown in bold. The last three reactions belong to the non-native substrates.

Annotation of the 6-phosphogluconate dehydrogenase (6PGDH) superfamily, despite including 12 enzyme families in Structural Classification of Proteins (SCOP) database (Andreeva *et al*., 2008) involved in many fundamental metabolic pathways (Fig. 1), represents a complicated challenge. At the sequence and structural levels, the superfamily is characterized by a broadly occurring G-X-G-X-X-G sequence motif, which is actually a feature of many dehydrogenases that use NAD(H)^+^/NADP(H)^+^ as cofactors, and a domain comprising the universal Rossmann fold. Annotation of enzymes belonging to this superfamily is further confounded by the broad range of chemically diverse substrates that this superfamily can convert (Mondal *et al*., 2010). For example, the NADH-dependent D-2-hydroxyacid dehydrogenases from the bacteria *Enterococcus faecalis* and *Lactococcus lactis* have been incorrectly annotated as ketopantoate reductases [KPR, Enzyme Commission (EC) 1.1.1.169, *panE* gene] (Wada *et al*., 2008; Chambellon *et al*., 2009).

Family 3 of this superfamily solely includes ketol-acid reductoisomerases (KARI, EC 1.1.1.86, *ilvC* gene). These are particularly interesting enzymes, as they perform two different reactions, and their substrate promiscuity is essential for the biosynthesis of the branched-chain amino acids, namely, valine, isoleucine and leucine. Previous enzyme mechanistic studies in *Escherichia coli*, *Pseudomonas aeruginosa* and plants (Dumas *et al*., 1994a,b; Biou *et al*., 1997; Ahn *et al*., 2003; Tyagi *et al*., 2005) have shown that the isomerization and the reduction activities of KARI can be separated and measured *in vitro*. Thus, KARI catalyses an alkyl migration followed by an NADP(H)^+^-dependent reduction. An implication of this is that the physiologically relevant substrates of KARI are 2-hydroxy-2-methyl ketobutyrate (2H2M3KB) in valine biosynthesis and 2-hydroxy-2-ethyl ketobutyrate (2H2E3KB) in isoleucine biosynthesis. Moreover, the reductase activity of KARI is exerted upon the intermediates 3-hydroxy-3-methyl-2-ketobutyrate (3H3M2KB) and 3-hydroxy-3-ethyl-2-ketobutyrate (3H3E2KB) respectively (Fig. 1). Therefore, KARI enzymes have at least four different substrates; however, only one EC number has been used to classify this enzyme.

Despite the large number of qualitative studies on promiscuous enzymes from different perspectives (O'Brien and Herschlag, 1999; Copley, 2003; Bornscheuer and Kazlauskas, 2004; Khersonsky *et al*., 2006; Hult and Berglund, 2007; Kim and Copley, 2007; Patrick *et al*., 2007; Khersonsky and Tawfik, 2010), there have been few efforts to systematically quantify this important property (Chakraborty and Rao, 2012). In 2008, Nath and colleagues published the index of substrate promiscuity, which is an entropy-based metric used to compare the promiscuous behaviour of enzymes. The promiscuity index uses information theory to describe catalytic efficiency of a set of enzymes towards various substrates. This metric is the probability that any given substrate will be the first one to be converted when an enzyme is simultaneously exposed to equal, low concentrations of all available substrates (Nath and Atkins, 2008; Nath *et al*., 2010). Moreover, this index, which ranges from 0 to 1, has two forms: a standard (*I*) and a weighted index (*J*). While *I* only takes into account the catalytic efficiency, *J* incorporates the chemical similarity of substrates, as given by a Tanimoto coefficient (Willet *et al*., 1998).

Here, we report the biochemical characterization of 10 enzymes annotated as KARIs from species belonging to the order *Actinomycetales*, renowned to include prolific producers of natural products, such as *Streptomyces*. *In vitro* results, using a total of eight chemically diverse substrates revealed a differential promiscuous behaviour, including: (i) reduction of pyrroline-5-carboxylate (P5C), a substrate chemically unrelated to the keto acids typically converted by KARIs and (ii) diversification or specialization of *ilvC* paralogues, which seemed to depend on their genomic and metabolic contexts. These observations guided *in vivo* genetic-based experiments, allowing for the discovery of a biosynthetic interlock between proline and the branched-chain amino acids in *Streptomyces*, as well as an assessment of the trade-off between *in vitro* and *in vivo* data for molecular functional annotation, by means of using substrate promiscuity indices.

## Results and discussion

### Chemical universe of the 6PGDH enzyme superfamily

Given that KARI, the central subject of this study, is a reductase, we arbitrary limited our analyses to the sub-set of the 6PGDH enzyme superfamily that are reductases (Fig. 1). This includes, in addition to family 3 (KARI), family 9 (KPR) and 10 (P5CR), and the enzyme 2-hydroxy-3-oxopropionate reductase (2H3OPR) of family 1 [hydroxyisobutyrate dehydrogenase (HBDH)-6PGDH]. KPR of family 9, an enzyme involved in pantothenate biosynthesis, reduces a hydroxyl keto acid into a dihydroxy acid, but without being preceded by an isomerization step as in KARI (Zheng and Blanchard, 2000). In contrast, the reductase activity of P5CR of family 10, involved in proline biosynthesis, catalyses a different reduction as reflected by the second digit of its EC number (i.e. 1.5.1.2). The bond being reduced by P5CR involves the nitrogen atom of pyrroline, rather than the oxygen of more frequently encountered carbonyl (Nocek *et al*., 2005). 2H3OPR of family 1, which also includes the dehydrogenases of 6-phosphogluconate and 3-hydroxyisobutyrate, catalyses the reduction of 2-hydroxy-3-oxopropionate to glycerate (Osipiuk *et al*., 2009).

To better understand the relationships between these reductases, we aimed to explore the chemical distribution of the native substrates of all enzymes of 6PGDH superfamily. We also analysed three keto acids that share chemical similarity with substrates of this superfamily, but which are not known to be the main substrate of members of this superfamily. This latter sub-set, which includes pyruvate (Pyr), hydroxypyruvate (HP) and methyl-acetoacetate (MAA), is referred to as non-native substrates. A modified list of descriptors published previously by Nath and Atkins (2008), including novel *ad hoc* descriptors for keto acids, was then obtained (Table S1). This took into account: (i) the position of the hydroxyl group (primary, secondary, tertiary alcohol), (ii) the nature of the alkyl groups (methyl, ethyl) and (iii) the position adopted by these alkyl groups.

**Figure 2:**
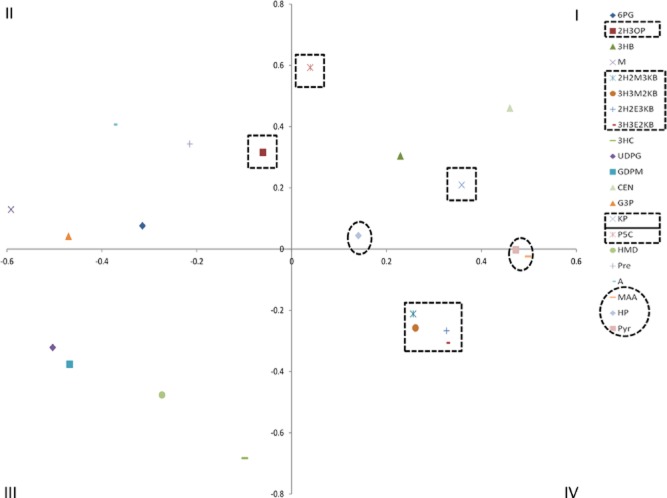
Chemical diversity of the 6PGDH superfamily. Two-dimentional representation of the chemical distance matrix using multidimensional scaling. Distribution represents normalized Tanimoto dissimilarity scores between each substrate. The *quadrants* are labelled with *Roman numerals.* Native substrates of reductases from 6PGDH superfamily are shown in dotted boxes. 2H2M3KB, 3H3M2KB, 2H2E3KB and 3H3E2KB cluster together in the fourth quadrant with non-native substrates (dashed circles) MAA and Pyr. Most distant P5C and HP are distributed in quadrant one, while 2H3OP is in quadrant two.

The result obtained after this analysis is a symmetrical distance matrix and can be displayed two-dimensionally by using multidimensional scaling. The resulting perceptual map is a graphic representation within a coordinate system of different features or distances of objects, in this case, substrates. In such a Cartesian view, distances between each substrate are represented as normalized chemical dissimilarity scores, which therefore lack units. Overall, the results shown in Fig. 2 highlight the sensitivity of the keyset of descriptors used to calculate the Tanimoto coefficient (Table S4). The result of this analysis shows that substrates are evenly distributed throughout all four quadrants and they seem to cluster in accordance to conserved functional groups.

Given that most enzyme families act upon chemically closely related substrates, it was unexpected to find that members of family 1 recognize substrates distributed throughout two quadrants. Such wide distribution may be due to differences in functional groups between these three substrates: 6-phosphogluconate has a phosphate group that is absent from the other two substrates; whereas 2-hydroxy-3-oxopropionate is a hydroxyacid semialdehyde and 3-hydroxyisobutyrate is a hydroxyacid that lacks a carbonyl group. Figure 2 also shows the distribution of the three non-native keto acids. MAA has a keto group prone to be reduced, as well as an akyl moiety prone to migration as in some native substrates. This substrate clusters together with Pyr, yet slightly away from HP, which is the only non-native substrate with a hydroxyl group.

### Selection and chemical synthesis of substrates

**Figure 3:**
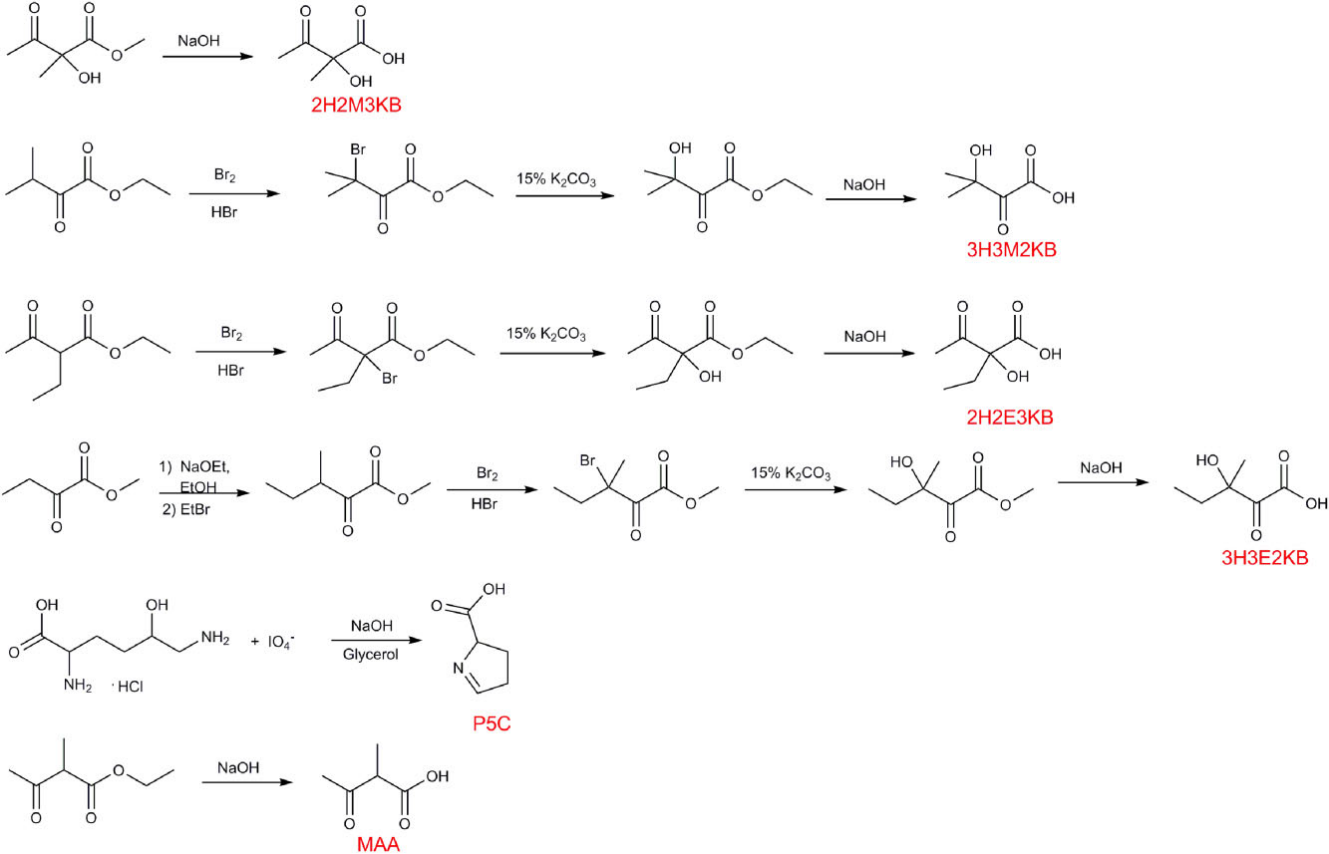
Chemical synthesis of substrates of KARI. Synthesis of 2-hydroxy-2-methyl-3-ketobutyrate (2H2M3KB); 3-hydroxy-3-methyl-2-ketobutyrate (3H3M2KB); 2-hydroxy-2-ethyl-3-ketobutyrate (2H2E3KB); 3-hydroxy-3-ethyl-2-ketobutyrate (3H3E2KB); Pyrroline-5-carboxylate (P5C); and 2-methylacetoacetate (MAA).

In addition to the substrates of KARI (2H2M3KB/3H3M2KB for valine and 2H2E3KB/3H3E2KB for isoleucine), the substrate of family 10, P5C, which is the direct precursor of proline, was selected. P5C is the most dissimilar substrate that is subject to reduction, and to our knowledge, it has never been tested as a KARI substrate, although this enzyme has been extensively biochemically characterized (Primerano and Burns, 1983; Dumas *et al*., 1994a,b; 1995; 2001; Biou *et al*., 1997; Ahn *et al*., 2003; Tyagi *et al*., 2005). Following adaptations from previously reported protocols (Williams and Frank, 1975; Chunduru *et al*., 1989; Tyagi *et al*., 2005), these substrates, as well as the non-native substrate MAA, were chemically synthesized as described in Fig. 3.

The substrate ketopantoate (KP), unfortunately, could not be included because its precursor for chemical synthesis, dihydro-4,4-dimethyl-2,3-furandione, is subject to strict banning policies where this work was performed. Furthermore, the substrate 2H3OP of 2H3OPR, as the sole reductase of the multi-enzyme family 1, could not be purified after synthesis. Given that 3H3OP and HP are tautomers, their separation is difficult. The previously catalytic parameters of the enzyme 3H3OPR from *Pseudomonas putida* and *Pseudomonas acidovorans*, where D-glycerate metabolism involving this enzyme was first described (Kohn and Jakoby, 1968), were actually measured in the presence of HP. Thus, use of HP was used as a substitute for 3H3OP. In total, eight ligands, including five native and three non-native substrates, belonging to two of the four quadrants of the Cartesian plane (Fig. 2), were experimentally characterized. The set of enzymes used for these experiments were selected as described in the following section.

### Selection and biochemical analysis of actinobacterial KARI homologues

**Figure 4:**
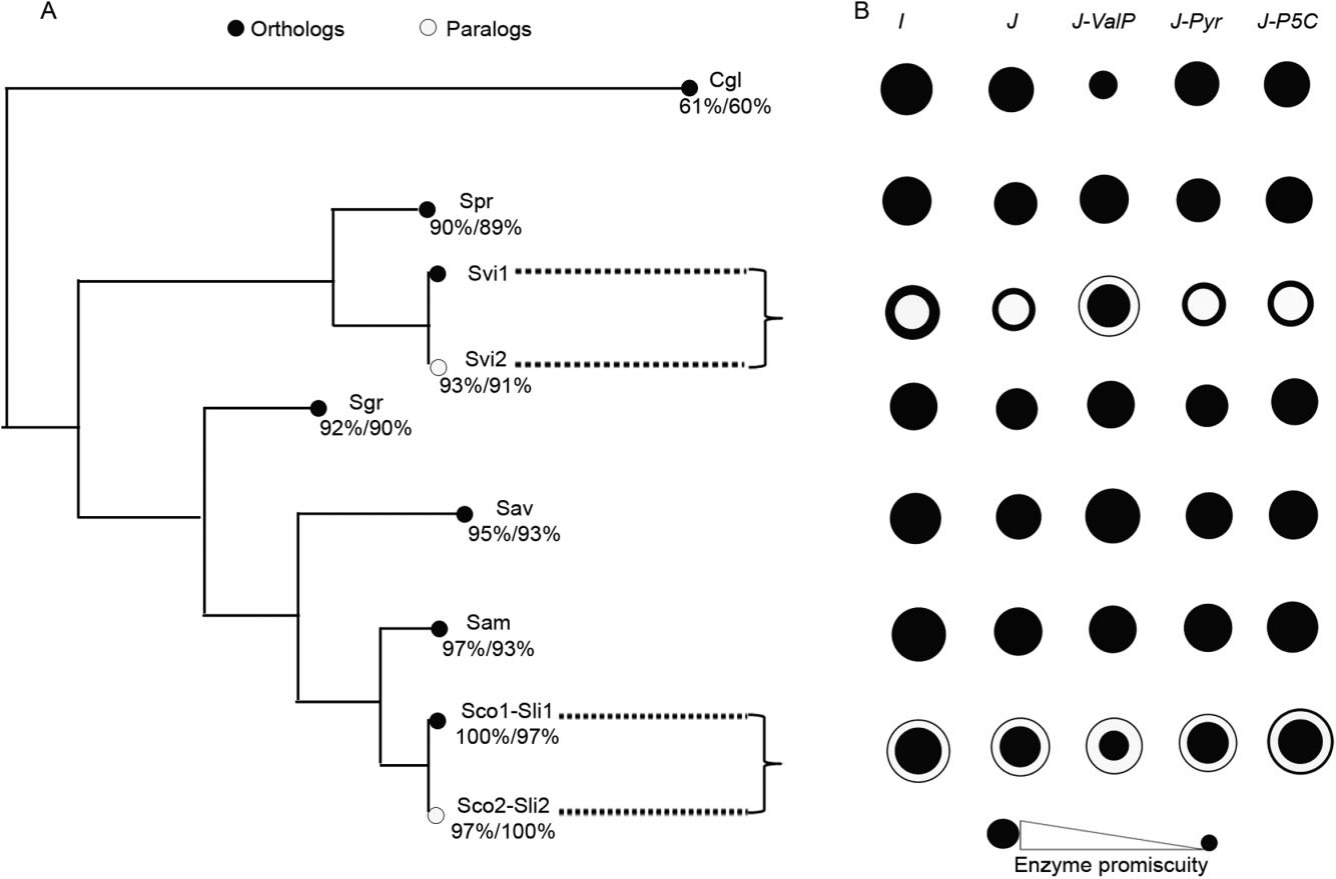
Promiscuous behaviour of phylogenetically related KARI homologues.A. Evolutionary relationships of KARI homologues based on RpoB and KARI phylogenies. Percentages provided are sequence identity calculated with respect to Sco1/Sco2.B. Substrate promiscuity indices *I* and *J* for orthologues are shown as black circles, other than paralogues that are shown as white circles. The two paralogous independent events investigated in this study are highlighted with a key. Names of enzymes are as in Table 1. Refer to text for further details.

After reconstruction of a phylogenetic tree using actinobacterial sequences of KARI homologues obtained from public databases, as well as sequences of the taxonomic marker RpoB (Fig. S1), around two dozen of KARI homologues were selected for functional analysis. Based on the phylogenetic distribution of the selected KARIs, covering different genera within the *Actinobacteria*, we decided to focus in the genus *Streptomyces*. The genomic coverage of this genus has witnessed a substantial increase, allowing for the identification of lineages where gene duplication events have occurred. A diagram that aims to recapitulate the evolutionary history of the selected *ilvC* homologues, including two independent paralogous events, is shown in Fig. 4A. As an out-group, the KARI enzyme from the actinobacterium *Corynebacterium glutamicum*, Cgl, was adopted. We selected Cgl as an out-group because this enzyme has been shown to compensate for the lack of a *panE* gene in this organism (Merkamm *et al*., 2003), and thus a physiologically relevant dual-substrate specificity (KPR and KARI activities) can be safely assumed.

The KARI homologues that could be successfully cloned for heterologous expression and purification purposes (Table S2) led to a final list of 10 enzymes. Nine of these enzymes belong to the genus *Streptomyces* and one to *C. glutamicum*. The only organism that was analysed that lacks a complete genome sequence is *Streptomyces viridifaciens*, although it has been proposed that its KARI homologue, Svi2, is the result of a gene duplication event (Garg *et al*., 2010). This is supported by the fact that this gene is part of an *ilvBNCE* operon that is next to the valanimycin biosynthetic gene cluster, which differs from the conserved *ilvBNC* operon – without *ilvE –* present in all of the available *Streptomyces* genomes (Fig. S2). The other paralogous relationship that was included is that of Sco1/Sco2 and Sli1/Sli2 from *S. coelicolor* and *S. lividans* respectively. Given that Sco1 and Sli1 are identical, and Sco2 and Sli2 are also identical, it is reasonable to conclude that this duplication must have occurred very recently but before speciation of these organisms. Thus, a single paralogous relationship for these four enzymes was considered.

Steady-state enzyme kinetic parameters were obtained for the 10 soluble KARI homologues, using both native (Table 1) and non-native (Table 2) substrates, and IlvC from *E. coli* (Eco) as a negative control for all substrates other than valine precursors (ValP = 2H2MKB and 3H3MKB) and isoleucine precursors (IleP = 2H2E3KB and 3H3E2KB), which served as positive controls. Overall, the *K_M_* and *k_cat_* parameters were found to be better for ValP than IleP. Highlighting this trend is Svi2 from *S. viridifaciens*, which has the highest *k_cat_*/*K_M_* of all enzymes, due to slightly better *K_M_* and *k_cat_* parameters for ValP, but yet the worst *K_M_* parameter for IleP. As a result, a 100-fold better catalytic efficiency (*k_cat_*/*K_M_*) for ValP can be seen. Thus, it is reasonable to conclude that Svi2 has a specialized function for the synthesis of valine, which serves as precursor for valanimycin, a natural product that has never been shown to incorporate isoleucine or leucine into its structure (Fig. S2). In fact, an amino acid decarboxylase encoded by the *vlmD* gene of the valanimycin biosynthetic gene cluster has been shown to be highly specific towards valine (Garg *et al*., 2010).

**Table 1:** Kinetic parameters of selected KARI homologues against native substrates

Substrate	Kinetic parameters^a^	Eco	Sco1	Sco2	Sli1	Sli2	Sam	Enzyme Sav	Sgr	Spr	Svi2	Cgl	P5CR
2H2M3KB	*k_cat_* (s^−1^)	2 ± 0.097	1.1 ± 0.2	0.24 ± 0.01	0.7 ± 0.59	0.28 ± 0.02	0.63 ± 0.5	0.24 ± 0.2	0.42 ± 0.04	3.6 ± 0.3	2 ± 0.07	3.2 ± 0.4	ND^b^
	*K_M_* (mM)	0.28 ± 0.03	1.6 ± 0.2	12 ± 0.8	1.2 ± 0.07	9.3 ± 0.8	2 ± 0.15	0.8 ± 0.09	1.2 ± 0.7	9 ± 0.7	1.8 ± 0.9	8 ± 0.9	ND^b^
	*k_cat_/ K_M_*(M^−1^ s^−1^)	7142 ± 689	687. ± 43	20 ± 3.2	583 ± 64	30 ± 3.8	315 ± 33	300 ± 39	350 ± 37	400 ± 38	1111 ± 115	400 ± 15	ND^b^
3H3M2KB	*k_cat_* (s^−1^)	3.1 ± 0.25	2.2 ± 0.15	0.3 ± 0.04	2.8 ± 0.3	0.4 ± 0.05	0.8 ± 0.1	1.2 ± 0.17	0.5 ± 0.06	5.2 ± 0.6	2.5 ± 0.3	0.9 ± 0.08	ND^b^
	*K_M_* (mM)	0.26 ± 0.03	3 ± 0.2	10 ± 0.11	4 ± 0.5	8.6 ± 0.9	4 ± 0.3	13 ± 0.9	8 ± 0.9	8.6 ± 0.9	5 ± 4.8	2.3 ± 0.25	ND^b^
	*k_cat_/ K_M_*(M^−1^ s^−1^	11923 ± 980	733 ± 7	30 ± 3.3	700 ± 71	45 ± 4	200 ± 54	520 ± 58	850 ± 90	530 ± 50	500 ± 48	391 ± 38	ND^b^
2H2E3KB	*k_cat_* (s^−1^)	3.5 ± 0.031	3.2 ± 0.2	0.39 ± 0.04	3.6 ± 0.4	0.12 ± 0.02	1.3 ± 0.09	0.4 ± 0.04	0.44 ± 0.03	0.18 ± 0.03	0.3 ± 0.02	0.9 ± 0.08	ND^b^
	*K_M_* (mM)	0.3 ± 0.028	67 ± 7	12 ± 1	71 ± 5	5 ± 0.3	6.7 ± 0.7	2.6 ± 0.3	2.5 ± 0.3	10 ± 0.9	25 ± 6	75 ± 5	ND^b^
	*k_cat_/ K_M_*(M^−1^ s^−1^)	11666 ± 1000	47 ± 4	32 ± 2	50 ± 0.4	48 ± 5	194 ± 20	153 ± 10	176 ± 12	18 ± 1	12 ± 1	12 ± 2	ND^b^
3H3E2KB	*k_cat_* (s^−1^)	3.8 ± 0.039	1.8 ± 0.2	0.8 ± 0.02	1.3 ± 0.04	0.9 ± 0.03	0.9 ± 0.1	0.8 ± 0.08	1 ± 0.05	1.4 ± 0.9	0.5 ± 0.03	0.18 ± 0.01	ND^b^
	*K_M_* (mM)	0.29 ± 0.03	45 ± 3	20 ± 3	50 ± 6	20 ± 1.9	20 ± 2	40 ± 4	35 ± 3	50 ± 6	50 ± 3	5 ± 1	ND^b^
	*k_cat_/ K_M_*(M^−1^ s^−1^)	13103 ± 1000	40 ± 3	40 ± 5	46 ± 3	46 ± 4	45 ± 4	20 ± 3	28 ± 2	28 ± 3	10 ± 0.9	36 ± 4	ND^b^
P5C	*k_cat_/ K_M_*(M^−1^ s^−1^)	ND^b^	2.4 ± 0.3	3 ± 0.4	3.7 ± 0.4	6.1 ± 0.6	5.5 ± 0.6	5.6 ± 0.5	2.4 ± 0.2	2.8 ± 0.3	3.8 ± 0.3	ND^b^	601 ± 70

a Kinetic parameters shown are means and standard errors of three enzymatic reaction.

b ND, activity not determined because it is below the limit of detection of the enzyme assay, which is *k_cat_*/*K_M_* = 0.000013 M^−1^s^−1^.

Enzymes nomenclature: Eco: KARI, *ilvC*, *Escherichia coli*; Sco1: KARI1, *ilvC1*, *Streptomyces coelicolor*; Sco2: KARI2, *ilvC2*, *Streptomyces coelicolor*: Sli1: KARI1, *ilvC1*, *Streptomyces lividans*; Sli2: KARI2, *ilvC2*, *Streptomyces lividans*; Sam: KARI, *ilvC*, *Streptomyces ambofaciens*; Sav: KARI, *ilvC*, *Streptomyces avermitilis;* Sgr: KARI, *ilvC*, *Streptomyces griseus*; Spr: KARI, *ilvC*, *Streptomyces pristinaespiralis*; Svi2: KARI2, *ilvC2*, *Streptomyces viridifaciens*; Cgl: KARI, *ilvC*-*panE*, *Corynebacterium glutamicum;* P5CR: P5CR, *proC*, *Streptomyces coelicolor*.

**Table 2:** Kinetic parameters of selected KARI homologues against non-native substrates

Substrate	Kinetic parameters^a^	Sco1	Sco2	Sli1	Sli2	Sam	Enzyme^c^ Sav	Sgr	Spr	Svi2	Cgl	P5CR
MAA	*k_cat_* (s^−1^)	1 ± 0.2	0.6 ± 0.05	0.675 ± 0.06	0.5 ± 0.05	1.2 ± 0.09	1.8 ± 0.1	1.05 ± 0.1	2 ± 0.2	2 ± 0.2	0.4 ± 0.03	ND^b^
	*K_M_* (mM)	50 ± 4	60 ± 5	45 ± 4	50 ± 4	40 ± 4	30 ± 2	35 ± 4	40 ± 5	50 ± 5	40 ± 1.5	ND^b^
	*k_cat_/ K_M_*(M^−1^ s^−1^)	20 ± 1.8	10 ± 1	15 ± 1.2	10 ± 0.9	30 ± 3	60 ± 7	30 ± 2.5	50 ± 6	40 ± 5	10 ± 1.3	ND^b^
HP	*k_cat_* (s^−1^)	ND^b^	0.5 ± 0.05	ND^b^	0.7 ± 0.06	1 ± 0.09	2 ± 0.2	1.5 ± 0.1	2 ± 0.2	2 ± 0.2	0.5 ± 0.04	ND^b^
	*K_M_* (mM)	ND^b^	40 ± 3	ND^b^	50 ± 5	50 ± 4	60 ± 5	30 ± 4	60 ± 5	50 ± 5	40 ± 4	ND^b^
	*k_cat_/ K_M_*(M^−1^ s^−1^)	ND^b^	60 ± 7	ND^b^	40 ± 3.5	20 ± 2.2	50 ± 5	40 ± 3.8	30 ± 3	50 ± 4	60 ± 3.9	ND^b^
Pyr	*k_cat_* (s^−1^)	2.8 ± 0.05	5.6 ± 0.5	6.7 ± 0.7	3 ± 0.2	6.3 ± 0.5	3.6 ± 0.4	1 ± 0.1	3 ± 0.2	5.7 ± 0.5	8.6 ± 0.9	ND^b^
	*K_M_* (mM)	950 ± 80	700 ± 70	1120 ± 110	1010 ± 100	1050 ± 99	1200 ± 110	1060 ± 120	1080 ± 100	1140 ± 112	1070 ± 118	ND^b^
	*k_cat_/ K_M_*(M^−1^ s^−1^)	3 ± 0.2	8 ± 0.7	6 ± 0.5	3 ± 0.2	6 ± 0.5	3 ± 0.3	1 ± 0.2	3 ± 0.3	5 ± 0.4	8 ± 0.7	ND^b^

a Kinetic parameters shown are means and standard errors of three enzymatic reaction.

b ND, activity not determined because it is below the limit of detection of the enzyme assay, which is *k_cat_*/*K_M_* = 0.000013 M^−1^s^−1^.

c Enzymes nomenclature: Eco: KARI, *ilvC*, *Escherichia coli*; Sco1: KARI1, *ilvC1*, *Streptomyces coelicolor*; Sco2: KARI2, *ilvC2*, *Streptomyces coelicolor*: Sli1: KARI1, *ilvC1*, *Streptomyces lividans*; Sli2: KARI2, *ilvC2*, *Streptomyces lividans*; Sam: KARI, *ilvC*, *Streptomyces ambofaciens*; Sav: KARI, *ilvC*, *Streptomyces avermitilis;* Sgr: KARI, *ilvC*, *Streptomyces griseus*; Spr: KARI, *ilvC*, *Streptomyces pristinaespiralis*; Svi2: KARI2, *ilvC2*, *Streptomyces viridifaciens*; Cgl: KARI, *ilvC*-*panE*, *Corynebacterium glutamicum;* P5CR: P5CR, *proC*, *Streptomyces coelicolor*.

The paralogues Sco2 and Sli2 show a 15-fold lower *k_cat_*/*K_M_* catalytic efficiency for ValP compared with their corresponding Sco1 and Sli1 paralogous partners. The trade-off of the kinetic parameters of Sco2 and Sli2 could be a sign of functional divergence, implying a yet-to-be discovered enzyme function. As in the case of other promiscuous reductases involved in the chlorophyll cycle (Ito and Tanaka, 2014), it could be that the dehydrogenation and not the reduction reaction is favoured. This would imply that Sco2 and Sli2 are better suited for catalysing the reverse reaction. This may be related to the fact that Sco1 and Sli1 are unable to reduce HP, which contradicts all other KARIs, including Sco2 and Sli2 (Table 2). These subtle functional differences become relevant in the context of the high sequence identity (97%) shared between Sco1 with Sco2, and Sli1 with Sli2, warranting further investigation.

All KARI homologues, moreover, could be saturated with the different substrates tested, with the exception of P5C (Table 1). The activity upon this substrate was found to be very low but present in all *Streptomyces* KARIs. The positive control for these experiments was P5CR or ProC from *S. coelicolor*. For the *Streptomyces* KARIs, therefore, only the catalytic efficiencies and not *K_M_* and *k_cat_* parameters determined independently could be obtained. Interestingly, we could not detect signs of conversion of P5C by Cgl, not even using highly sensitive *in vivo* complementation assays based in high copy number plasmids and an *E. coli proC* minus proline auxotroph (see below)*.* This could be attributable to the fact that Cgl has a physiologically relevant KPR activity (Merkamm *et al*., 2003), implying a P5CR and KPR activity trade-off during the course of evolution.

### *In vivo* genetic-based analysis of functional predictions

P5CR has been postulated to be a remote homologue of KARI, as these enzymes share 19% at the sequence level and they show strong structural similarities (Nocek *et al*., 2005). As a quick method to look for P5CR activity, we used an *E. coli* proline auxotrophic mutant that lacks the *proC* gene (Baba *et al*., 2006). The inability of this strain to grow in M9 minimal media without proline could be rescued with all of the different KARI homologues, previously cloned into a pASK plasmid derivative suitable for complementation assays (Table S2), other than Cgl from *C. glutamicum*. Moreover, since all of these KARI homologues do complement an *ilvC* mutant from the same collection, this result supports the proposed trade-off between the P5CR and KPR activities of Cgl during the course of evolution.

We then aimed to construct a set of mutants in *S. coelicolor*, useful for *in vivo* complementation assays. Previously, we have shown that a *proC* minus mutant of *S. coelicolor*, termed WP101, is prototrophic (Barona-Gomez and Hodgson, 2006). Based on our biochemical analysis, it is tempting to speculate that the remaining P5CR activity of strain WP101 could be related to the promiscuous P5CR activity of Sco1 and/or Sco2. Construction of a triple *proC*, *ilvC1* and *ilvC2* mutant, ideally unmarked to avoid polar effects (*proC* and *ilvC1* seem to be part of operons), was therefore attempted. After an unsuccessful comprehensive screening to isolate double cross-over recombination events, or marker excision using a phage display method (Khodakaramian *et al*., 2006), we concluded that strong counter-selection for mutation of *ilvC1* in the absence of *proC* and *ilvC2* is operating. This counter-selection was overcome, however, by the use of two different resistant markers. Using as genetic background, a *proC::scar* unmarked mutant, the streptomycin (*aadA)* and apramycin [*aac(3)IV*] resistance cassettes were used to replace the *ilvC1* and *ilvC2* genes respectively.

The *S. coelicolor* mutants constructed in this study, as well as their growth requirements, are shown in Table S3. The *S. coelicolor ilvC1* and *ilvC2* double mutant, as expected, is auxotrophic for valine, leucine and isoleucine, while the triple mutant, in addition to being auxotrophic to these amino acids, is also auxotrophic for proline. To further analyse these mutants, five *ilvC* genes representative of different genetic backgrounds and catalytic efficiencies were cloned into a modified version of the pAV11B plasmid called pAV11B_FBG (Jyothikumar *et al*., 2012) (Table S2). This plasmid drives gene expression from a tetracycline-inducible *tcp830* promoter that can be strongly induced with anhydrotetracycline, and thus poor enzyme activities can be revealed. The selected *ilvC* genes include those coding for the proteins Sco1 and Sco2, as well as Svi2, as representatives of paralogous enzymes with different promiscuous behaviour; the *ilvC* gene from *S. griseus* as an example of a gene encoding a non-duplicated KARI and the *ilvC* gene of *C. glutamicum* as the sole member analysed here that lacks P5CR activity.

**Figure 5:**
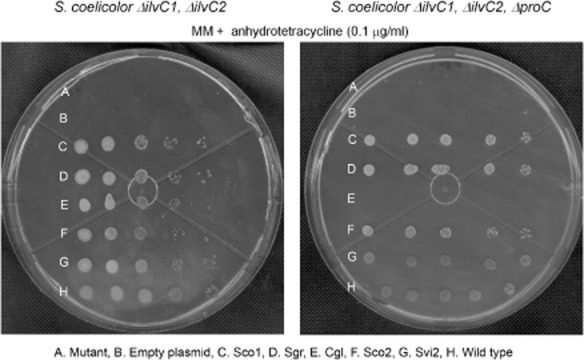
Functional *in vivo* analysis of selected KARIs. Complementation assays in *S. coelicolor* double (*ΔilvC1, ΔilvC2*) and triple (*ΔilvC1, ΔilvC2, ΔproC*) mutants in *Streptomyces* minimal medium (MM) supplemented with anhydrotetracycline (0.1 μg ml^−1^). Picture was taken after 4 days of growth. All strains other than A and H, i.e. from B to G, are the corresponding *S. coelicolor* mutant [marked as A: double *ilvC* mutant, left panel; triple *ilvC* and *proC* mutant, right panel] complemented with (B) empty plasmid, (C) Sco1, (D) Sgr, (E) Cgl, (F) Sco2 and (G) Svi2 (Names of enzymes are as in Table 1). H refers to *S. coelicolor* wild-type strain.

All *ilvC* genes, including Cgl, rescued growth of the *S. coelicolor* double mutant, as expected. Induction of expression with different anhydrotetracycline concentrations only reduced growth time for the case of the complemented strain with the Cgl-containing construct. In contrast, exactly under the same conditions, Cgl failed to rescue growth of the triple mutant, which is in agreement with the fact that a *C. glutamicum proC* mutant is a proline auxotroph (Ankri *et al*., 1996). Moreover, all of the *Streptomyces* enzymes did complement equally well the triple mutant (Fig. 5). Interestingly, the differential promiscuous behaviour of the paralogues Sco1, Sco2 and Svi2 was not reflected by these complementation assays.

Thus, we conclude, first, that promiscuous activities *in vivo*, even if small, are usually enough to support growth, something that has been previously broadly reported (Kim and Copley, 2007; Patrick *et al*., 2007) and second, that evolutionary trade-offs could drive or restrain enzyme promiscuity, even if chemically speaking reactions are actually feasible. These two conclusions provide insights into the future development of enzyme promiscuity indices, as further discussed below.

### Assessment of indices of substrate promiscuity

Standard *I* and weighted *J* indices of substrate promiscuity were calculated for the ten KARI homologues and the eight substrates that were experimentally tested (Table 3). In addition to the experimentally based calculations, for some cases where either the enzyme or the substrate could not be obtained, theoretical estimations were performed. These estimations were based on conservative assumptions considering physiological expectations. On one hand, as the substrate of KP could not be obtained and a physiologically relevant KPR activity has been demonstrated (Merkamm *et al*., 2003), we assumed that Cgl has a catalytic efficiency for KP reduction in the same order of magnitude than that detected for ValP. On the other hand, for the remaining enzymes from *Streptomyces*, we estimated low KPR catalytic efficiencies (20-fold lower with respect to ValP), as these would be similar to the scenario previously reported for KARI in *Salmonella typhimurium*, which has a *panE* gene (Primerano and Burns, 1983)*.* Also, we assumed that the central KARI of *S. viridifaciens*, i.e. the central metabolic paralogue of Svi2, has a catalytic efficiency for all substrates similar to the mean value of the parameters found in all KARI enzymes from *Streptomyces*.

**Table 3:** Enzyme substrate promiscuity indices

Enzyme^b^	Standard Index of substrate promiscuity (*I*)^a^^c^				Weighted Index of substrate promiscuity (*J*)^a^^c^			
	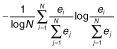				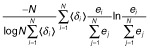			
	I	I-ValP	I-Pyr	I-P5C	J	J-ValP	J-Pyr	J-P5C
Cgl	0.71	0.57	0.74	0.75	0.61	0.39	0.61	0.69
Spr	0.67	0.71	0.70	0.70	0.59	0.67	0.60	0.64
Svi1	0.67	0.62	0.70	0.70	0.59	0.59	0.60	0.63
Svi2	0.47	0.81	0.49	0.49	0.42	0.79	0.43	0.46
Sgr	0.65	0.66	0.68	0.67	0.57	0.64	0.58	0.61
Sav	0.70	0.77	0.73	0.73	0.62	0.74	0.64	0.67
Sam	0.74	0.66	0.77	0.77	0.66	0.64	0.66	0.70
Sco2	0.83	0.75	0.83	0.83	0.77	0.73	0.76	0.83
Sli2	0.80	0.71	0.83	0.83	0.75	0.70	0.75	0.78
Sco1	0.64	0.42	0.67	0.67	0.56	0.40	0.57	0.61
Sli1	0.64	0.51	0.67	0.67	0.56	0.48	0.56	0.60
Sco_P5CR	0	0	0	0	0	0	0	0

a Both indices range between 0 and 1.

b Enzyme nomenclature is as in Table 1.

c *N*, number of substrates; e, catalytic efficiency; δ_i_ = δ_ij_ / δ_set_, δ_ij_, the mean Tanimoto distance from a member i to all the other members in the set; δ_set_, the overall set dissimilarity.

The calculated *I* and *J* indices, including some perturbations where certain substrates were omitted as further discussed in the following paragraph, are shown in Table 3. Furthermore, these data are also shown in a graphical fashion in Fig. 4B, where circles are used to represent the degree of substrate promiscuity of the enzyme. Single copies are shown as black circles, whereas paralogues are shown as white circles. Overall, we note that *I* and *J* indices are quite similar, as expected from the fact that all enzymes have high catalytic efficiencies for more than one substrate, e.g. ValP. High catalytic efficiency would therefore mask the fact that the substrates tested are chemically quite different. In other words, the contribution of poor activities upon chemically diverse substrates is not captured by either of these indices. Thus, we hypothesized that removal of ValP, which includes the substrates with the better enzyme kinetic parameters, might expose the contribution of low activities, as well as the contribution of chemical diversity. Likewise, removal of a similar substrate to ValP, e.g. Pyr, or a chemically dissimilar substrate, e.g. P5C, when poor enzyme kinetic parameters are recorded, would lead to marginal changes. For this purpose, we recalculate the Tanimoto distances (Table S6) as well as *I* and *J*, but omitting P5C, ValP and Pyr (Fig. 4B).

As KP and ValP are highly similar, when the latter is removed, Cgl appears to behave as a non-promiscuous enzyme, with specificity for KP, as expected. This perturbation also makes Sco1 and Sli1 appear less promiscuous, which would be equivalent to becoming specialized for IleP. Interestingly, this perturbation unmasked the specialized nature of Svi2 for ValP, as it seems to become a promiscuous enzyme with the remaining substrates, for which none has good enzyme kinetic parameters. The effect of removing ValP is such that the behaviour of the paralogues in *S. viridifaciens* is even inverted. In contrast, removal of Pyr or P5C only marginally affects the possible behaviour of all enzymes, the exception being the case of Sco2 and Sli2 for P5C. In this case, the apparent increase of the promiscuity of these enzymes relates to the fact that none of the remaining substrates, which are chemically quite dissimilar to P5C, is converted at a high catalytic efficiency. Indeed, the values of the standard *I* index for Sco2 and Sli2 (0.83 and 0.80 respectively) becomes virtually identical to the values obtained for the *J-P5C* perturbation (0.83 and 0.78 respectively).

Our results disagree with previous analyses using highly efficient non-specific enzymes, such as gluthatione S-transferase, proteases and cytochrome P450, which were concluded to be highly promiscuous (Nath and Atkins, 2008; Nath *et al*., 2010). In our case, the *I* and *J* indices only partially paint the portrait of the promiscuous nature of KARI enzymes. Although the detoxifying enzymes have evolved broad substrate specificity, KARI enzymes seemed to have evolved low secondary promiscuous activities, as previously defined (O'Brien and Herschlag, 1999; Copley, 2003; Khersonsky *et al*., 2006; Khersonsky and Tawfik, 2010). Even when the fingerprint-based approaches used to calculate Tanimoto distances capture important elements of chemical similarity, it is clear that when low enzyme activities are considered, the model does not describe reality.

The fact that enzyme promiscuity has been shown to be essential for survival of the organism upon certain conditions or genetic backgrounds (Kim and Copley, 2007; Patrick *et al*., 2007) raises questions about the utility of *I* and *J* indices at their current state, as they fail to describe secondary and low enzyme promiscuous activities. This assessment therefore opens the possibility to further develop enzyme promiscuity metrics that would include other features of the substrates and enzymes, such as three-dimensional structural architecture, molecular mass, hydrophobicity and electrostatic charges (Ferro and Bredow, 2010), as well as the evolutionary history of enzymes.

## Experimental procedures

### Synthesis of KARI substrates

ValP and IleP substrates were obtained by alkaline hydrolysis of the corresponding esters by 1.1 equiv of potassium hydroxide followed by addition of 1 M Tris, pH 7.5. Ester of 2H2M3KB is commercially available, the rest of the esters were obtained as follows: synthesis of ethyl 3H3M2KB, the ester of 3H3M2KB, was prepared from bromination and hydroxylation of ethyl 3-methyl-2-ketobutyrate as described by (Chunduru *et al*., 1989). Ethyl 3H3E2KB, the ester of 3H3E2KB, was prepared using ethyl-2-ethylacetoacetate as precursor. Methyl 2-hydroxy-2-ethyl-3-ketobutyrate, the ester of 2H2E3KB, was prepared using methyl-2-oxobutanoate as the initial precursor, which required an alkylation step (Brändström, 1959), using sodium hydroxide in ethanol and ethyl bromide. Resulting compound methyl 2-ethyl-3-ketobutyrate was subjected to bromination and hydroxylation as described earlier. After purification, esters were characterized by ^1^H nuclear magnetic resonance (NMR) and ^13^C NMR. Synthesis, purification and quantification of P5C were made as described by Williams and Frank (1975). All precursors, as well as Pyr and HP, were purchased from Sigma-Aldrich®.

### Calculation of Tanimoto distances

The modified list of descriptors used to help distinguish our substrates is shown in Table S1. We calculate the Tanimoto distance (Willet *et al*., 1998) by using a perl script, available upon request, which creates a bit vector (0 for absent descriptor, 1 if it is present) for each substrate. The final output of this script is a distance matrix (Table S4), which was used for construction of a perceptual map showing the relationships between the substrates using permat version 11.8a.

### Protein expression and purification

All *ilvC* genes, including that of *E. coli* as a negative control, were cloned into pET28a from Novagene (Table S2). *Escherichia coli* strain BL21 Star (DE3) (Invitrogen) was used as host for expression. Luria–Bertani media with 0.5 mM of isopropyl β-D-1-thiogalactopyranoside (IPTG), at an OD_600nm_ of 0.7 and 17°C for 12 h in agitation (200 r.p.m.), was used. Purification of enzymes was performed using Ni-NTA column VivaPure maxiprep MC (Santorius Stedim Biotech) at 4°C. As equilibration buffer, 20 mM Tris-HCl with 500 mM NaCl, pH 7.9, was used. Elution of proteins from the Ni-NTA column was done at 150 mM of imidazole, pH 7.9. Proteins were dialysed against several changes of Tris HCl buffer, pH 8, and concentrated using an Amicon Ultra centrifugal filter (Millipore). Final concentration was determined using Bio-Rad protein assay dye reagent.

### Steady-state enzyme kinetics

Initial velocities (*v*_0_) were determined at 25°C*.* The reaction buffer used contained 0.1 M Tris-HCl (pH 8), 10 mM MgCl_2_, 0.22 mM NADP(H)^+^, and different concentrations of the substrates (0–100 mM) up to saturation of the enzymes were used, in a final volume of 100 μl. The enzyme concentration for each assay was determined by measuring the rate of the reaction at a constant substrate concentration of 100 mM. The NADP(H)^+^ oxidation was followed at 340 nm in a Cary spectrophotometer 100 Bio, after enzymes were added to the reaction mix. Kinetic constants *K_M_* and *k_cat_* were calculated by non-linear regression fit of the initial velocity data to the Michaelis–Menten equation, and the limit of detection was calculated to be *k_cat_*/*K_M_* = 0.000013 M^−1^ s^−1^.

### Bacterial molecular genetics

Oligonucleotides used in this study are shown in Table S5. Strains, plasmids and cosmids used in this study are listed in Table S2. All genes were amplified by polymerase chain reaction and cloned with standard methods, except for *ilvC2* of *S. viridifaciens* (Garg *et al*., 2010), which was commercially synthesized (GenScript). *Streptomyces* strains were grown using mannitol soya medium and minimal medium media previously reported (Kieser *et al*., 2000). Antibiotics (Sigma-Aldrich®) were added at the following concentrations: ampicillin (50 μg ml^−1^), apramycin (50 μg ml^−1^), chloramphenicol (25 μg ml^−1^), kanamycin (50 μg ml^−1^), nalidixic acid (25 μg ml^−1^) and hygromycin (50 μg ml^−1^). Gene disruptions in *S. coelicolor* were done as previously (Gust *et al*., 2012). A *proC*^−^ mutant from the Keio collection (Baba *et al*., 2006) and the *ilvC* genes cloned into pASK plasmid were used for P5CR complementation in *E. coli*. Transformed cells were grown, washed and plated on M9 media. For complementation assays in *S. coelicolor*, the growth requirements of the double and triple mutants (*proC::scar ilvC1::aadA, ilvC2::aac(3)IV*) were obtained using MM supplemented with the relevant amino acids at a concentration of 7.5 μg ml^−1^. Mutants were obtained after conjugation, using a methylation-deficient *E. coli* host ET12567 / pUZ8002 (Gust *et al*., 2012) and pAV11B_FBG (a modified version of pAV11B; Jyothikumar *et al*., 2012) bearing the *ilvC* genes. The same number of spores was plated for the complementation assays.

### Substrate promiscuity indices

Standard *I* and weighted *J* indices (ranging between 0 and 1) were obtained using the equations 4 and 5 described in Nath and Atkins (2008). In order to recalculate the indices with different scenarios, we obtained the distance matrix with the Perl script omitting the valine precursors, P5C and Pyr (Table S6).

## Supporting Information

**Fig. S1.** Phylogenetic reconstruction using sequences of RpoB and KARI homologues from *Actinobacteria*. **Fig. S2.** A. Genome context analysis of *ilvC* genes in *Streptomyces*. C. Biosynthetic pathway for valanimycin. **Table S1.** Descriptor keyset used for obtaining Tanimoto distance of KARI substrates. **Table S2.** Plasmids generated in this study by sub-cloning. **Table S3.** Growth requirements of *S. coelicolor* mutants. **Table S4.** Tanimoto distance matrix calculated for all substrates of 6PGDH superfamily constructed using descriptors showed in Table S1. **Table S5.** Oligonucleotides used in this study for PCR amplification and disruption of the genes *ilvC1*, *ilvC2*, *proC* in *S. coelicolor*. **Table S6.** Recalculated matrix of Tanimoto distances matrix of substrates used to calculate promiscuity indices from KARI homologues including some perturbations.
