# Supplementary material for: Molecular annotation of ketol-acid reductoisomerases from Streptomyces reveals a novel amino acid biosynthesis interlock mediated by enzyme promiscuity
Source: Microb Biotechnol. 2014 Oct 9;8(2):239–52. doi: 10.1111/1751-7915.12175 (PMC4353338; doi:10.1111/1751-7915.12175)
Supplement: Supplementary file 1 [file mbt20008-0239-sd1.zip › mbt212175-supp-0001-figureS1-2 ; tableS1-3,5.docx]

**Supplementary Information for “Molecular annotation of ketol-acid reductoisomerases from *Streptomyces* reveals a novel amino acid biosynthesis interlock mediated by enzyme promiscuity”**

Karina Verdel-Aranda^1^, Susana T. López-Cortina^2^ David A. Hodgson^3^ & Francisco Barona-Gómez^1*^

**Figure S1. Phylogenetic tree reconstruction using PhyML.** Maximum likelihood trees were constructed with approximate likelihood ratio test (aLRT ) support of the nodes and LG as substitution model obtained using ProtTest 2.4 server A) Phylogenetic reconstruction using sequences of RpoB and B) KARI homologues from *Actinobacteria*.

**(A)**

**(B)**

**(B)**

**Figure S2.** A) **Genome context analysis of *ilvC* genes in *Streptomyces***. A) Valanimycin biosynthetic gene cluster of *S. viridifaciens,* including the duplicated *ilvBNCE* operon (first line); *ilvC* gene duplication in *S. coelicolor* and *S. lividans* (second line); and single copy *ilvC* gene in all other *Streptomyces* analyzed. B) Functions of deduced genes for valanimycin and branched chain amino acid biosynthesis. C) Biosynthetic pathway for valanimycin ([Garg and Parry, 2010](#_ENREF_1))*.*

*
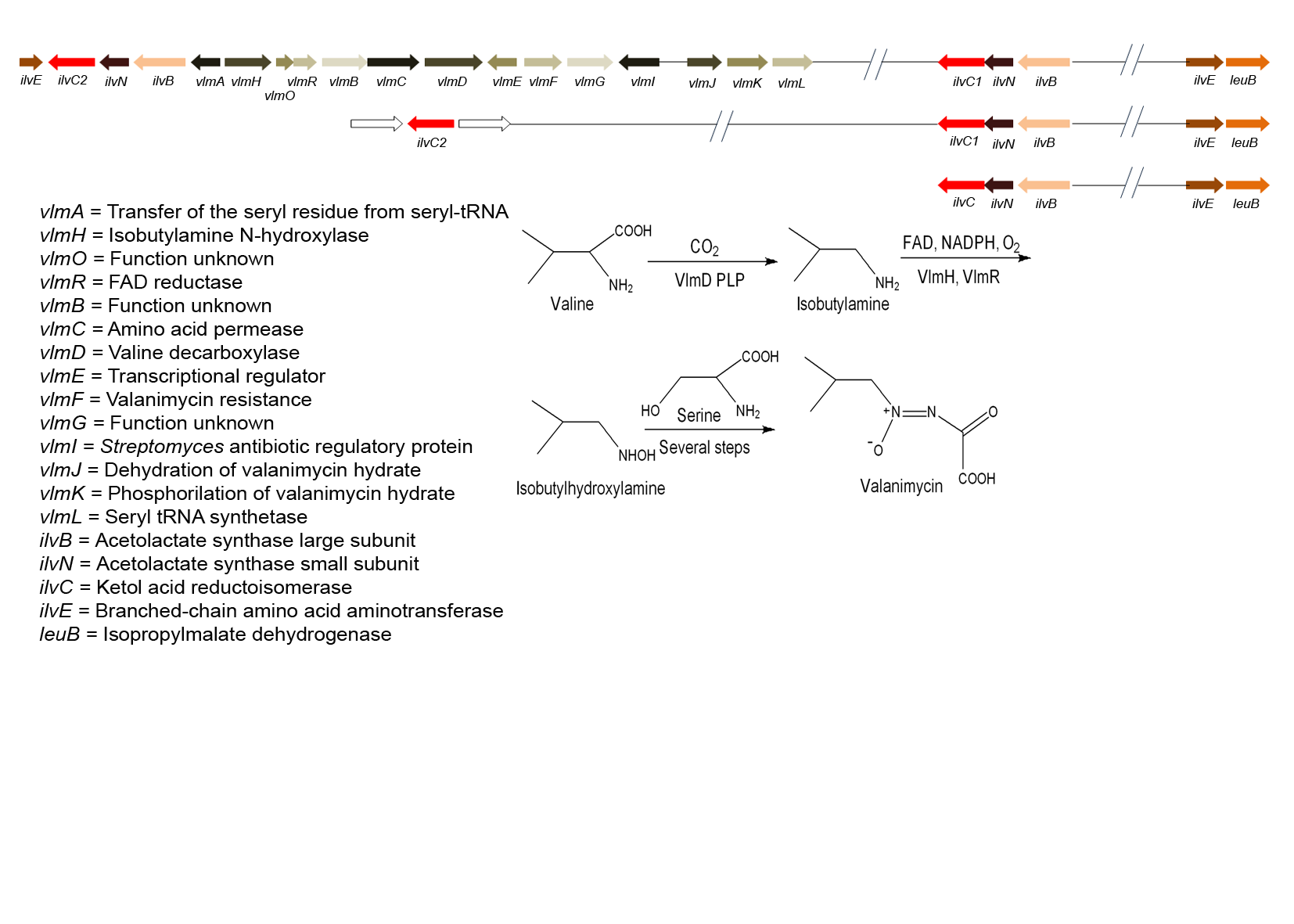
*

**(B)**

**(C)**

**(A)**

**Table S1.** Descriptor keyset used for obtaining Tanimoto distance of KARI homologues substrates.

| Bit number | Descriptor |
| --- | --- |
| 1 | Contains at least one carbon atom |
| 2 | Contains at least one heteroatom (excluding carbon and hydrogen) |
| 3 | Contains at least one oxygen atom |
| 4 | Contains at least one nitrogen atom |
| 5 | Contains at least one sulfur atom |
| 6 | Contains at least one phosphorus atom |
| 7 | Contains at least one halide |
| 8 | Contains at least one chloride |
| 9 | Contains at least one bromide |
| 10 | Contains at least one iodide |
| 11 | Contains at least one fluoride |
| 12 | Contains at least 3 heteroatoms |
| 13 | Contains at least 5 heteroatoms |
| 14 | Contains at least 8 heteroatoms |
| 15 | Carries a charge |
| 16 | Contains at least 10 heavy atoms (excluding hydrogen) |
| 17 | Contains at least 15 heavy atoms (excluding hydrogen) |
| 18 | Contains at least 25 heavy atoms (excluding hydrogen) |
| 19 | Contains at least 40 heavy atoms (excluding hydrogen) |
| 20 | Contains at least one double bond |
| 21 | Contains at least one carbon-carbon double bond |
| 22 | Contains at least one triple bond |
| 23 | Contains at least one carbon-carbon triple bond |
| 24 | Contains at least one ring |
| 25 | Contains at least 2 rings |
| 26 | Contains at least 3 rings |
| 27 | Contains a 5-membered ring |
| 28 | Contains a 6-membered ring |
| 29 | Contains a 7-membered ring |
| 30 | Contains a ring with 8 or more members |
| 31 | Contains a steroid nucleus |
| 32 | Contains at least one aromatic ring |
| 33 | Contains a 5-membered aromatic ring |
| 34 | Contains a 6-membered aromatic ring |
| 35 | Contains a 7-membered aromatic ring |
| 36 | Contains an aromatic ring with 8 or more members |
| 37 | Contains at least 2 aromatic rings |
| 38 | Contains at least 3 aromatic rings |
| 39 | Contains an aliphatic chain |
| 40 | Contains an aliphatic chain at least 3 carbon atoms in length |
| 41 | Contains an aliphatic chain at least 5 carbon atoms in length |
| 42 | Contains an aliphatic chain at least 7 carbon atoms in length |
| 43 | Contains an aliphatic chain at least 9 carbon atoms in length |
| 44 | Contains an aliphatic chain at least 11 carbon atoms in length |
| 45 | Contains an aliphatic chain at least 13 carbon atoms in length |
| 46 | Contains an aliphatic chain at least 15 carbon atoms in length |
| 47 | Contains at least one carbonyl group |
| 48 | Contains at least two carbonyl groups |
| 49 | Contains at least one carboxylic acid |
| 50 | Contains at least two carboxylic acids |
| 51 | Contains at least one acyl halide |
| 52 | Contains at least two acyl halides |
| 53 | Contains at least one aldehyde |
| 54 | Contains at least two aldehydes |
| 55 | Contains at least one alcohol |
| 56 | Contains at least two alcohols |
| 57 | Contains at least one ketone |
| 58 | Contains at least two ketones |
| 59 | Contains at least one peroxide |
| 60 | Contains at least two peroxides |
| 61 | Contains at least one epoxide |
| 62 | Contains at least two epoxides |
| 63 | Contains at least one nitro group |
| 64 | Contains at least two nitro groups |
| 65 | Contains at least one amino group |
| 66 | Contains at least two amino groups |
| 67 | Contains at least one amide |
| 68 | Contains at least two amides |
| 69 | Contains at least one imino group |
| 70 | Contains at least two imino groups |
| 71 | Contains at least one imido group |
| 72 | Contains at least two imides |
| 73 | Contains at least one nitroso group |
| 74 | Contains at least two nitroso groups |
| 75 | Contains at least one cyanide |
| 76 | Contains at least two cyanides |
| 77 | Contains at least one isocyanide |
| 78 | Contains at least two isocyanides |
| 79 | Contains at least one cyanate |
| 80 | Contains at least two cyanates |
| 81 | Contains at least one isocyanate |
| 82 | Contains at least two isocyanates |
| 83 | Contains at least one isothiocyanate |
| 84 | Contains at least two isothiocyanates |
| 85 | Contains at least one α,β-unsaturated carbonyl group |
| 86 | Contains at least two α,β-unsaturated carbonyl groups |
| 87 | Contains at least one sulfate |
| 88 | Contains at least two sulfates |
| 89 | Contains at least one sulfone |
| 90 | Contains at least two sulfones |
| 91 | Contains at least one thiol |
| 92 | Contains at least two thiols |
| 93 | **Contains a primary alcohol** |
| 94 | **Contains a secondary alcohol** |
| 95 | **Contains a tertiary alcohol** |
| 96 | **Contains at least an alkyl group** |
| 97 | **Contains a methyl group** |
| 98 | **Contains an ethyl group** |
| 99 | **Contains a propyl group** |
| 100 | **Contains at least two alkyl groups** |

**Table S2.** Plasmids generated in this study by sub-cloning

| Plasmid | Characteristics | *In vivo* complementation of P5CR / KARI activities ^a^ | Reference or source |
| --- | --- | --- | --- |
| pET28a | T7lac promoter, N-terminal His tag, Km^R^ |  | Novagene |
| pET28a_ *Sco_ilvC1* | *Sco_ilvC1* cloned into *Nde*I and *Hind*III of pET28a |  | This work |
| pET28a_ *Sco_ilvC2* | *Sco_ilvC2* cloned into pET28a |  | This work |
| pET28a_ *Sli_ilvC1* | *Sli_ilvC1* cloned into pET28a |  | This work |
| pET28a_ *Sli_ilvC2* | *Sli_ilvC2* cloned into pET28a |  | This work |
| pET28a_*Sam_ilvc* | *Sam_ilvC* cloned into pET28a |  | This work |
| pET28a_*Sav_ilvc* | *Sav_ilvC* cloned into pET28a |  | This work |
| pET28a_ *Sgr_ilvC* | *Sgr_ilvC* cloned into pET28a |  | This work |
| pET28a_ *Spr_ilvC* | *Spr_ilvC* cloned into pEt28a |  | This work |
| pET28a_ *Svi_ilvC2* | *Svi_ilvC2* cloned into pEt28a |  | This work |
| pET28a_ *Cgl_ilvC* | *Cgl_ilvC* cloned into pET28a |  | This work |
| pASK | Derivative of pASK_IBA3plus (w/*Nde*I site) | -/- | Unpublished, Iba - Lifesciences |
| pASK_*Sco_ilvC1* | *Sco_ilvC1* cloned into pASK | +/+ | This work |
| pASK_*Sco_ilvC2* | *Sco_ilvC2* cloned into pASK | +/+ | This work |
| pASK_*Sli_ilvC1* | *Sli_ilvC1* cloned into pASK | ++ | This work |
| pASK_*Sli_ilvC2* | *Sli_ilvC2* cloned into pASK | +/+ | This work |
| pASK_*Sam_ilvC* | *Sam_ilvC* cloned into pASK | +/+ | This work |
| pASK_*Sav_ilvC* | *Sav_ilvC* cloned into pASK | +/+ | This work |
| pASK_*Sgr_ilvC* | *Sgr_ilvC* cloned into pASK | +/+ | This work |
| pASK_*Spr_ilvC* | *Spr_ilvC* cloned into pASK | +/+ | This work |
| pASK_*Svi_ilvC2* | *Svi_ilvC2* cloned into pASK | +/+ | This work |
| pASK_*Cgl_ilvC* | *Cgl_ilvC* cloned into pASK | -/+ | This work |
| pAV11b_FBG | pMS82 with *tcp830* promoter and *tetris*; *hyg^R^* modified for cloning with *Nde*I and *Hind*III | -/- | Unpublished |
| pAV11b_FBG *_Sco_ilvC1* | *Sco_ilvC1* cloned into pAV11b_FBG | +/+ | This work |
| pAV11b_FBG_ *Sco_ilvC2* | *Sco_ilvC2* cloned into pAV11b_FBG | +/+ | This work |
| pAV11b_ FBG*_Sgr_ilvC* | *Sgr_ilvC* cloned into pAV11b_FBG | +/+ | This work |
| pAV11b_FBG *_Svi_ilvC2* | *Svi_ilvC2* cloned into pAV11b_FBG | +/+ | This work |
| pAV11b_ FBG*_Cgl_ilvC* | *Cgl_ilvC* cloned into pAV11b_FBG | -/+ | This work |
| pAV11b_ FBG*_Sco_proC* | *Sco_proC* cloned into pAV11b_FBG | +/- | This work |
| Cosmid SC8D9.26 | PCR template for *ilvC1* disruption |  | John Innes Centre |
| Cosmid SCE7.04c | PCR template for *proC* disruption |  | John Innes Centre |
| Cosmid SC9A4.16c | PCR template for *ilvC2* disruption |  | John Innes Centre |

**^a^** Constructs that were not evaluated are shown as empty cells. Experiments using pASK are in *E. coli* strains, whereas experiments using pAV11b_FBG are in *Streptomyces* strains

**Table S3.** Growth requirements of *S. coelicolor* mutants on solid minimal media supplemented with selected sources of amino acids.

| Strain | Genotype | Growth on MM | Growth on MM with I, V, L & P ^a^ | Growth on MM with I, V & L | Growth on MM with CAA ^a^ |
| --- | --- | --- | --- | --- | --- |
| *S. coelicolor* | Wild type | Normal ^b^ | Normal | Normal | Normal |
| **WP101** | *proC::aac(3)IV* | Normal | Normal | Normal | Normal |
| **WP102** | *proC::scar* | Normal | Normal | Normal | Normal |
| **WILV103** | *ilvC1::aac(3)IV* | Slightly Reduced | Normal | Normal | Normal |
| **WILV101** | *ilvC2::aac(3)IV* | Normal | Normal | Normal | Normal |
| **WILV102** | *ilvC2::scar* | Normal | Normal | Normal | Normal |
| **WILVPK101** | *proC::scar, ilvC1::aac(3)IV* | Slightly Reduced | Normal | Slightly Reduced | Normal |
| **WILVPK102** | *proC::scar, ilvC2::aac(3)IV* | Normal | Normal | Normal | Normal |
| **WILVK101** | *ilvC1::aac(3)IV, ilvC2::scar* | No Growth | Very Reduced | Normal | Normal |
| **WILVPK101** | *proC::scar. ilvC1::strp, ilvC2:: aac(3)IV* | No Growth | Very Reduced | No Growth | Normal |

**^a^** I, isoleucine; L, leucine; V, valine; P, proline; CAA, casaminoacids.

**^b^** Normal represents wild-type growth rate.

**Table S5.** Oligonucleotides used in this study for PCR amplification and disruption of the genes *ilvC1*, *ilvC2*, *proC* in *S. coelicolor* (REDIRECT) ([Gust *et al.*, 2003](#_ENREF_2))

| Gene(s) | **PCR template** | **Primers (restriction sites *Nde*I-*Hin*dIII)** | Vector |
| --- | --- | --- | --- |
| Sco_proC | *S. coelicolor* chromosomal DNA | GGAATTCCATATGGGACGCCCGCTTCTACTT  CCGATAAGCTTTCAGTCCTTGGTGCC | pASK*, pET28a, pAV11B_FBG |
| Sco_ilvC1 | *S. coelicolor* chromosomal DNA | GGAATTCCATATGAGCAAGCTCGAACTC  CCGATAAGCTTCTACGCCTCTTCGTCGAC | pASK*, pET28a, pAV11B_FBG |
| Sco_ilvC2 | *S. coelicolor* chromosomal DNA | GGAATTCCATATGGCAAGCTCGAACTC  CCGATAAGCTTTCACGCCTCTTCGTCGAC | pASK*, pET28a, pAV11B_FBG |
| Sli_ilvC1 | *S. lividans 1326* chromosomal DNA | GGAATTCCATATGGTGGCCGAGCTGTTCTAC  CCGATAAGCTTTTCAACCAGCGGGGCAACCAAC | pASK*, pET28a, pAV11B_FBG |
| Sli_ilvC2 | *S. lividans 1326* chromosomal DNA | GGAATTCCATATGTGATTATTCCGGCATTAGA  CCGATAAGCTTTGGAAGCGGTGATATCGTAGAA | pASK*, pET28a, pAV11B_FBG |
| Sam_ilvC | *S. ambofaciens* chromosomal DNA | GGAATTCCATATGATTATTCCGGCATTAGA  CCGATAAGCTTTGGAAGCGGTGATATCGTAGAACA | pASK*, pET28a, pAV11B_FBG |
| Sav_ilvC | *S. avermitilis* chromosomal DNA | GGAATTCCATATGGTGGCCGAGCTGTTCTACG  CCGATAAGCTTTTTACGCCTCTTCGTCGTTC | pASK*, pET28a, pAV11B_FBG |
| Sgr_ilvC | *S. griseus* chromosomal DNA | GGAATTCCATATGGTGGCCGAGCTGTTCTACG  CCGATAAGCTTCTAGGCCTCTTCGTCGTTC | pASK*, pET28a, pAV11B_FBG |
| Cglu_ilvC | *C. glutamicum* chromosomal DNA | GGAATTCCATATGGTGGCCGAGCTGTTCTACG  CCGATAAGCTTCTAGGCCTCTTCGTCGTTC | pASK*, pET28a, pAV11B_FBG |
| Red_ilvC1_Fw | pIJ773-pIJ774 | GTGGGACCGAACACCTGCACACAAGGAGAGAACCCAGTGATTCCGGGGATCCGTCGACC |  |
| Red_ilvC1_Rv | pIJ773-pIJ774 | CGCCCGGCGCGCCGGGCGCCCCCGCGGGGCAAAGACCTATGTAGGCTGGAGCTGCTTC |  |
| Red_ilvC2_Fw | pIJ773-pIJ774 | ATCCCCGCTCGGGGAGTTCTCCGAAAGGTGTGTCTCATGATTCCGGGGATCCGTCGACC |  |
| Red_ilvC2_Rv | pIJ773-pIJ774 | GCGCCGCCGTGGCGCGCCCACCGGCCTGCCCGGATGTCATGTAGGCTGGAGCTGCTTC |  |
| Red_proC_Fw | pIJ773-pIJ774 | ATGACCCCGGACCACGCGCACACCCCGGAGGGCACCGAAATTCCGGGGATCCGTCGACC |  |
| Red_proC_Rv | pIJ773-pIJ774 | TCAGTCCTTGGTGCCGGTGGCCAGTTCGCGGCTGCGGTCTGTAGGCTGGAGCTGCTTC |  |

Garg, R.P., and Parry, R.J. (2010) Regulation of valanimycin biosynthesis in *Streptomyces* *viridifaciens*: characterization of VlmI as a *Streptomyces* antibiotic regulatory protein (SARP). *Microbiology* **156**: 472-483.

Gust, B., Challis, G.L., Fowler, K., Kieser, T., and Chater, K.F. (2003) PCR-targeted *Streptomyces* gene replacement identifies a protein domain needed for biosynthesis of the sesquiterpene soil odor geosmin. *Proc Natl Acad Sci*U S A **100**: 1541-1546.
